# Endoscopic thyroidectomy to minimize light exposure of an erythropoietic protoporphyria patient: A case report

**DOI:** 10.1016/j.ijscr.2022.107227

**Published:** 2022-05-20

**Authors:** Tomo Ishisaka, Takuya Noda, Yuzo Shimode, Morimasa Kitamura, Hiroyuki Tsuji

**Affiliations:** Department of Head and Neck Surgery, Kanazawa Medical University, Japan

**Keywords:** Video-assisted neck surgery (VANS), Erythropoietic protoporphyria, Photosensitivity, Endoscopic thyroidectomy

## Abstract

**Introduction and importance:**

Erythropoietic protoporphyria is a rare form of cutaneous porphyria that presents with photosensitivity. During surgery, light shielding is required to prevent acute attacks due to photosensitivity. In this study, we report a case of successful endoscopic thyroidectomy using video-assisted neck surgery (VANS) method for a thyroid tumor in a patient with erythropoietic protoporphyria.

**Case presentation:**

A 32-year-old woman with a history of erythropoietic protoporphyria and an enlarged left thyroid gland was referred to our hospital. The referring physician had noted a left lobe tumor of the thyroid gland with a maximum diameter of 5 cm. Computed tomography and ultrasonography showed the tumor shape as smooth and well-defined. A lobectomy of the thyroid gland was performed using the VANS method through a 3-cm incision on the skin. Surgical light was not used and exposure to ambient light was minimized by covering the patient's neck with a drape. No acute attack of porphyria or photosensitivity was observed during the surgery or the follow-up. Postoperative complications such as asphyxia, blood loss, or nerve palsy were absent. The patient was discharged 5 days after the surgery.

**Clinical discussion:**

While the VANS method is known to have mainly cosmetic advantages, this case demonstrates that it is also a method that can minimize the exposure of the patient's skin to light with using an additional drape.

**Conclusion:**

The VANS method should be considered as an option for thyroidectomy in patients with protoporphyria because it can reduce the exposure to light during surgery.

## Highlights

• A case of thyroid tumor with erythropoietic protoporphyria
• Exposure of patient's skin to light should be minimized.
• The VANS method can be chosen to reduce light exposure during surgery.

## 1 Introduction

Porphyria is a rare genetic disease caused by an abnormality of the *heme synthase* gene and is classified as hepatic or erythropoietic depending on the location of the overproduction of porphyrin bodies [1]. It can also be classified into acute or cutaneous depending on the clinical symptoms. Erythropoietic protoporphyria is one of the cutaneous forms of porphyria that presents with photosensitivity [2]. In addition to photosensitivity, it also causes osteochondral defects and hemolytic anemia among other porphyria-specific serious symptoms. During surgery of a patient with erythropoietic protoporphyria, precautions against acute attacks resulting from photosensitivity are necessary. In case of endoscopic thyroidectomy, it is possible to avoid the use of surgical lights, thus reducing the patient's exposure to light. Here we report a case of endoscopic thyroidectomy using the video-assisted neck surgery (VANS) method [3] for a thyroid tumor in a patient with erythropoietic protoporphyria. This report has been reported in line with the current SCARE 2020 criteria [4].

## 2 Presentation of case

A 32-year-old woman had congenital erythropoietic protoporphyria, inherited from her father. She had consulted a dermatologist at another hospital, and through that treatment, her protoporphyrin levels decreased, so she ended her treatment. After that, the patient herself noticed a swelling of an enlarged left thyroid gland and visited her primary care doctor. A tumor of 2-cm diameter in the left lobe of her thyroid gland was noted by ultrasonography. The tumor was monitored by ultrasonography for 2 years, during which it had grown to a maximum diameter of 5 cm. A fine-needle aspiration biopsy was performed, but it could not be determined whether the tumor was benign or malignant. Because of the increasing size of the tumor and our department's extensive experience in thyroidectomy, the patient was referred to us. She had no previous history of surgery.

**Fig. 1:**
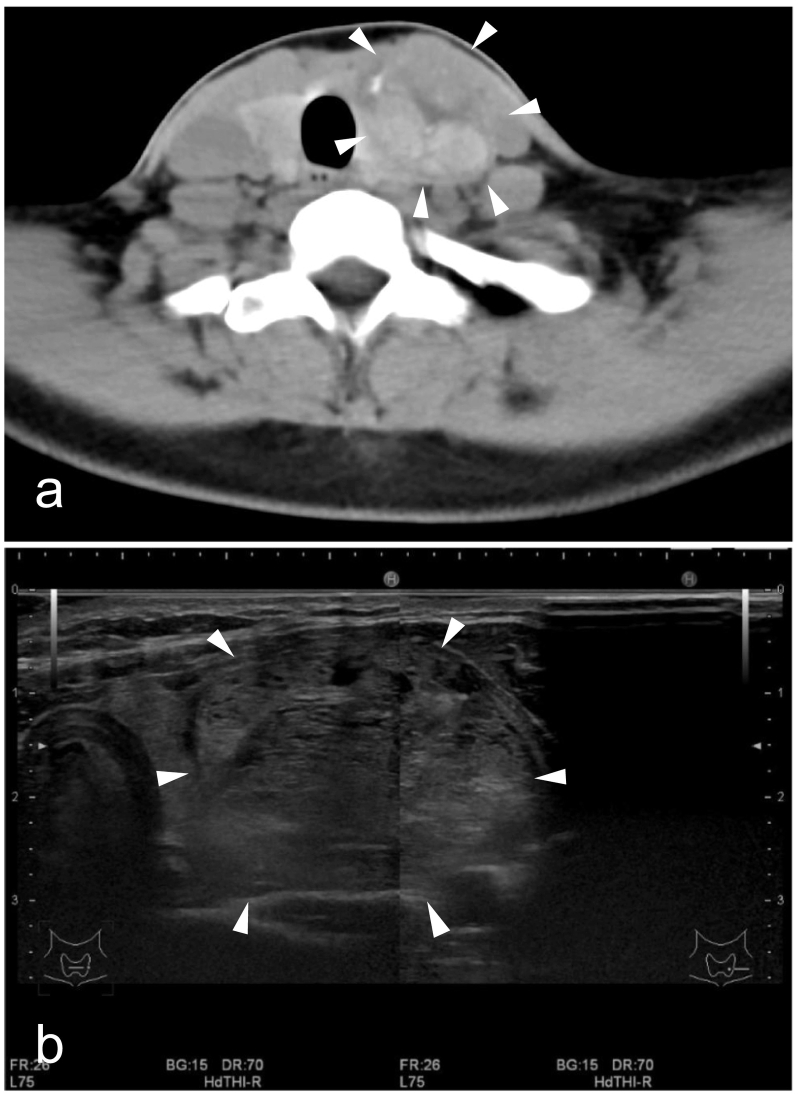
(a) Computed tomography image of a mass lesion (indicated by the arrowheads) with mixed low and high absorption profile in the left lobe of the thyroid gland. (b) Ultrasound image of the well-defined, smooth, and large tumor mass (indicated by the arrowheads) measuring 51 × 31 × 19 mm.

At our department, palpation revealed a large, elastic, and soft mass in the left thyroid gland. There was no laryngeal abnormality, and her bilateral vocal cords were mobile. Preoperative computed tomography images showed a mixed hypo- to hyperdense mass lesion in the left lobe of the thyroid gland (Fig. 1a). Ultrasonography revealed a large, smooth, and well-defined tumor measuring 51 × 31 × 19 mm (Fig. 1b). As the patient had erythropoietic protoporphyria, her consulting dermatologist recommended dimming the surgical lights as much as possible and covering her skin with a drape to minimize light exposure. Based on these factors and our extensive experience [5], [6], an endoscopic thyroidectomy using the VANS method was considered to be a rational choice. The patient provided consent to this surgery.

**Fig. 2:**
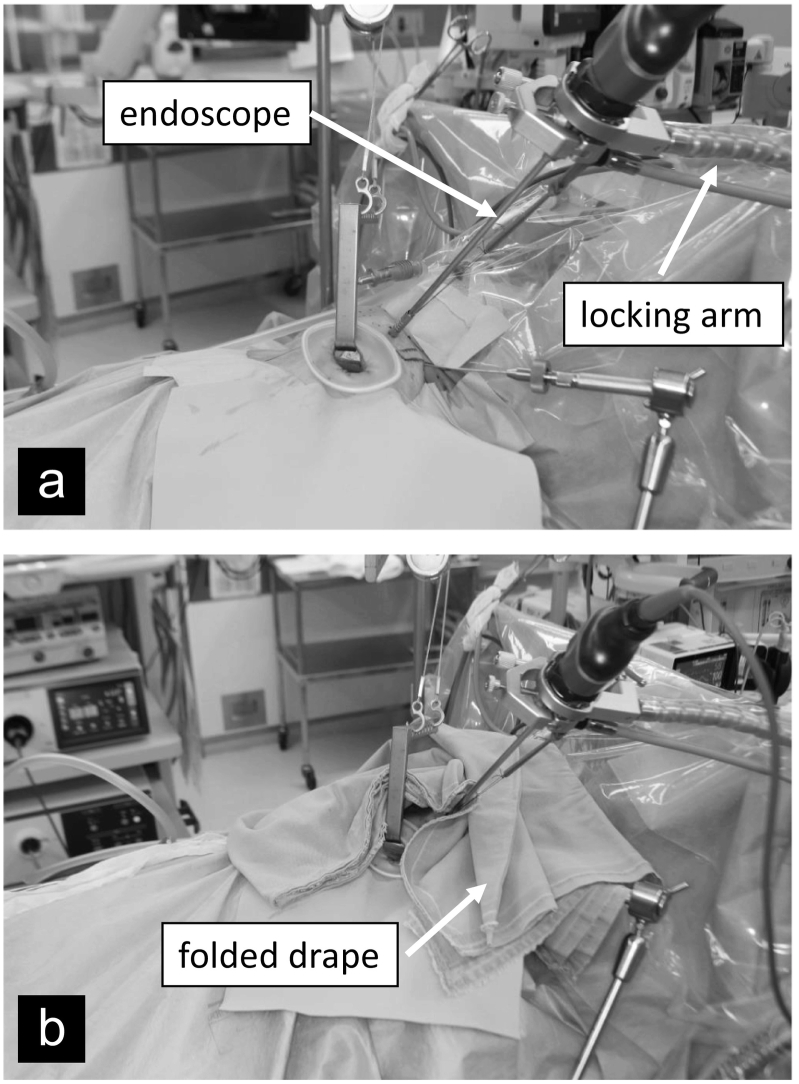
(a) Usual setting of the VANS method in our department: A rigid endoscope was inserted through a 5-mm camera port incision on the lower neck of the affected side and fixed to a locking arm. (b) A folded drape was used during thyroidectomy to protect the exposed skin of the patient's neck from light.

**Fig. 3:**
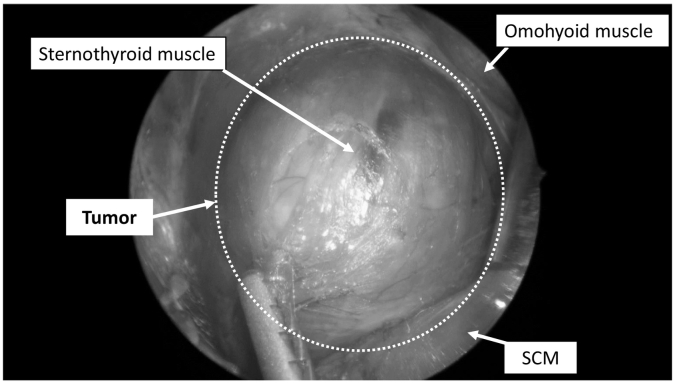
Endoscopic view of the tumor in the left lobe of the thyroid gland: The sternocleidomastoid and omohyoid muscles are pulled with a muscle hook to expose the tumor, which is present beneath a sternothyroid muscle. SCM: sternocleidomastoid muscle.

The patient was placed in the supine position with her neck extended using a shoulder pillow as in conventional thyroidectomy. Fig. 2a shows the usual setting of the VANS method in our department. No surgical light was used, and a folded drape was used to protect the exposed skin of the patient's neck from ambient light (Fig. 2b). An experienced surgeon performed the surgery. A 3-cm incision was made on the skin along the Langer dermatome line, approximately two transverse finger-lengths below the clavicle. A skin flap was created under the platysma muscle in direct view using only a headlight, without the surgical light. A skin-lifting hook (Mist-less VANS retractor set; Hakko, Japan) and a wire retractor (Kent traction device; Takasago Medical Industry, Japan) were used to suspend the skin flap. As seen in Fig. 2a, a rigid endoscope (Karl Storz, Japan) was inserted through a 5-mm camera port incision on the lower neck of the affected side and fixed to a locking arm (System JB, Japan). Then the sternocleidomastoid and omohyoid muscles were pulled with a muscle hook (Sonne, Japan) to expose the tumor (Fig. 3). A lobectomy of the thyroid gland was performed with attention to recurrent laryngeal nerve palsy. Postoperative histopathological examination confirmed that the tumor was an adenomatous nodule.

Postoperatively, the patient experienced no complications such as asphyxia or blood loss due to bleeding, hoarseness of voice or dysphagia due to recurrent laryngeal nerve palsy, high pitch dysphonia due to superior laryngeal nerve palsy, or sensory disturbance at the incision site. The drain was removed on the second postoperative day, all stitches were removed on the fifth postoperative day, and she was discharged. The number of hospitalization days in case of the VANS method was the same as that of the conventional endoscopic surgery.

Incidentally, as a treatment for erythropoietic protoporphyria, the patient used sunscreen from the first day of hospitalization according to her dermatologist's instructions. The patient was also assigned a room that blocked direct sunlight. No acute attacks of porphyria or photosensitivity were observed during the hospitalization. The dermatologist informed us that the patient's blood protoporphyrin level before surgery was 2898 μg/dLRBC (normal, 30–86 μg/dLRBC).

## 3 Discussion

Erythropoietic protoporphyria is one of the most common diseases in which exposure to light should be avoided. During surgery of patients with erythropoietic protoporphyria, measures should be taken against acute attacks due to photosensitivity to the surgical lights. Endoscopic or laparoscopic and abdominal surgery of short duration (less than 1.5 h) usually do not cause phototoxic damage [7]. Factors contributing to acute attacks include drugs, stress, starvation, and hormones. There are also many contraindications to these drugs [8]. As a countermeasure, we believe that drug administration and anesthesia management are necessary during the perioperative period [9]. In addition, since it is believed that photosensitivity is caused by light with a wavelength around 410 nm [10], the surgical lights act as a trigger. This can be prevented by using an optical filter that effectively blocks wavelengths in the 410 nm region [11] when using surgical lights, but the procedure is complicated by the need to measure the wavelength intraoperatively [12]. Therefore, it would be more desirable if there were a way to avoid the use of surgical lights.

The VANS method is highly useful from the viewpoint of cosmetic appearance because the incision is smaller and the postoperative wound is inconspicuous compared to conventional neck incision surgery performed without the use of an endoscope [3]. In Japan, thyroidectomy for malignant tumors, in addition to benign goiter and Graves' disease, is covered by the public health insurance. In addition, the VANS method is characterized by the fact that no insufflation gas is used and the working space is secured by suspending the skin valve. The present case report demonstrates that the VANS method can be successfully performed without the use of surgical lights. This adds to the advantages of choosing the VANS method for thyroidectomy in patients who should avoid exposure to light. In other words, while reducing operative time and using optical filters have been the main measures for light exposure so far [7], [12], the VANS method can be recognized as a promising alternative. We believe that head and neck surgeons should consider this method for thyroidectomy in patients with erythropoietic protoporphyria, as it is unlikely to cause acute photosensitivity attacks. However, caution must be exercised against protoporphyrin accumulation in the liver that can potentially cause severe hepatic damage [13]. Future clinical trials should focus on the effect of thyroidectomy using the VANS method on the extent of hepatic damage.

## 4 Conclusion

The VANS method is a frequently used technique in thyroidectomy, mainly because of its cosmetic advantages. We were able to perform thyroidectomy using the VANS method safely and successfully in a patient with erythropoietic protoporphyria. This procedure should be considered as a first-choice option for thyroidectomy in patients with protoporphyria because it minimizes the exposure of the patient's skin to light.

## Sources of funding

The authors have received no financial support for this study.

## Ethical approval

For this type of study, ethics approval was waived by the Kanazawa Medical University Medical Research Ethics Committee.

## Consent

Written informed consent was obtained from the patient for publication of this case report and any accompanying images.

## CRediT authorship contribution statement

Tomo Ishisaka: Participated in the surgery, data collection, case analysis, and writing of the manuscript. Takuya Noda: Participated in the surgery and collected the data. Yuzo Shimode: Analyzed the case data. Morimasa Kitamura: Supervised and validated the case data. Hiroyuki Tsuji: Perfoming the surgery, conceptualization and supervision.

## Research registration

Not applicable.

## Guarantor

Tomo Ishisaka, MD, MBBS.

## Provenance and peer review

Not commissioned, externally peer-reviewed.

## Declaration of competing interest

No benefits, in any form, have been received or will be received from a commercial party related directly or indirectly to the subject of this article.

## References

[1] Karim Z., Lyoumi S., Nicolas G., Deybach J.C., Gouya L., Puy H. (2015). Porphyrias: a 2015 update. Clin. Res. Hepatol. Gastroenterol..

[2] Ioi H., Suzuki S., Numabe H., Kawashima H., Shimura M. (2017). Clinical, pathological, and genetic study of three cases of erythropoietic protoporphyria diagnosed in childhood. J. Tokyo Med. Univ..

[3] Shimizu K., Akira S., Tanaka S. (1998). Video-assisted neck surgery: endoscopic resection of benign thyroid tumor aiming at scarless surgery on the neck. J. Surg. Oncol..

[4] Agha R.A., Franchi T., Sohrabi C., Mathew G., for the SCARE Group (2020). The SCARE 2020 guideline: updating consensus Surgical CAse REport (SCARE) guidelines. Int. J. Surg..

[5] Noda T., Okano K., Kobayashi Y., Nomura T., Shimode Y., Tsuji H. (2020). Experience of continuous intraoperative nerve monitoring in video assisted neck surgery [in Japanese]. J JAES JSTS.

[6] Noda T., Ishisaka T., Okano K., Kobayashi Y., Shimode Y., Tsuji H. (2021). Experience with the use of intraoperative continuous nerve monitoring in video-assisted neck surgery and external cervical incisions. Laryngosc. Investig. Otolaryngol..

[7] Kondo M., Nakayama K., Yano Y. (1999). Porphyrias and drugs [in Japanese]. Porphyrins.

[8] Weksler B.B., Gilman S. (2007). Neurobiology of Disease.

[9] Sasaki Y., Motoyama S., Okuyama M., Maruyama K., Ogawa J. (2005). A case of surgery for thoracic esophageal cancer with acute intermittent porphiria [in Japanese]. Jpn. J. Gastroenterol. Surg..

[10] Lecha M., Puy H., Deybach J.C. (2009). Erythropoietic protoporphyria. Orphanet J. Rare Dis..

[11] Takahashi R., Hirai I., Murayama S., Takeshita A., Watanabe T., Fukumoto T. (2013). Experience of cholecystectomy in a porphyria patient with photosensitivity [in Japanese]. J. Jpn. Biliary Assoc..

[12] Wahlin S., Srikanthan N., Hamre B., Harper P., Brun A. (2008). Protection from phototoxic injury during surgery and endoscopy in erythropoietic protoporphyria. Liver Transpl..

[13] Sasaki H. (1990). Progress in Liver Disease Research VI [in Japanese].

